# Associations of nutritional status and dietary habits with the development of female infertility. A case–control study

**DOI:** 10.3389/fnut.2024.1476784

**Published:** 2024-10-09

**Authors:** Laura Martín-Manchado, Antonio Manuel Moya-Yeste, Miriam Sánchez-Sansegundo, José Antonio Hurtado-Sánchez, Regina Andrea Gil-Miralles, José Tuells, Ana Zaragoza-Martí

**Affiliations:** ^1^Department of Nursing, Faculty of Health Sciences, University of Alicante, Alicante, Spain; ^2^Gynaecology and Obstetrics Service, Hospital IMED de Levante, Benidorm, Spain; ^3^Department of Health Psychology, Faculty of Health Sciences, University of Alicante, Alicante, Spain; ^4^Gynaecology and Obstetrics Service, Marina Baixa Health Department, Alicante, Spain; ^5^Alicante Institute for Health and Biomedical Research (ISABIAL-FISABIO Foundation), Alicante, Spain; ^6^Department of Community Nursing, Preventive Medicine, Public Health and History of Science, University of Alicante, Alicante, Spain

**Keywords:** nutritional status, dietary habits, female infertility, Mediterranean Diet, red meat

## Abstract

**Introduction:**

Female infertility is a multifactorial condition influenced by lifestyle and dietary factors. Understanding the relationship between nutritional status, dietary habits, and infertility could provide insights for targeted interventions.

**Methods:**

A case-control study was conducted in health centers and hospitals in Alicante, Spain. The study included 60 infertile and 30 fertile women aged 18–40, selected through consecutive sampling. Data on body composition and dietary intake were collected and analyzed.

**Results:**

Infertile women exhibited significantly lower muscle mass (*p* = 0.005) and larger hip circumference (*p* = 0.034) compared to fertile women. Additionally, a significant association was found between high red meat consumption and an increased risk of female infertility (*p* = 0.011).

**Discussion:**

These results suggest that body composition and dietary habits, particularly muscle mass and red meat intake, play a key role in female fertility. Interventions aimed at improving muscle mass, reducing localized body fat, and limiting red meat consumption may enhance fertility outcomes. Further longitudinal research is needed to confirm these findings across diverse populations.

## 1 Introduction

Infertility is defined as the inability of a couple to achieve pregnancy after 12 months of regular, unprotected intercourse (1). According to a recent report by the World Health Organization (WHO), around 17.5% of adults experience infertility (2). Therefore, this condition represents a serious public health problem with profound emotional, psychological, and social repercussions (1). As the incidence of infertility continues to rise, understanding its causes and risk factors becomes a crucial area of research (2).

Regarding the etiological factors of female infertility, various modifiable lifestyle factors, in addition to the aging process, have been identified. For example, smoking, high caffeine and alcohol consumption, chronic stress, and prolonged exposure to environmental pollutants adversely affect female reproductive health (3, 4). Similarly, nutritional status and dietary habits appear to be linked to the development of conditions and/or factors involved in female infertility (3, 4).

Concerning nutritional status, it is known that both excess body weight and malnutrition lead to ovarian function alterations, resulting in increased female infertility (3, 5). In cases of malnutrition, chronic energy deficiency and the lack of essential nutrients are associated with the suppression of the hypothalamic–pituitary-gonadal axis, which affects the release of gonadotropin-releasing hormone (5). This suppression triggers a cascade of inhibitory effects, including reduced gonadotropin secretion, slower follicular development, and decreased synthesis of gonadal steroid hormones (5). On the other hand, in women without ovulation disorders, overweight and obesity are also associated with reproductive problems (6). A key metabolic disruption in obesity is insulin resistance (IR), a condition where the body’s cells do not respond effectively to insulin, resulting in elevated blood glucose and insulin levels. Both IR and hyperinsulinemia (excess insulin in the blood) are characteristic of metabolic syndrome and polycystic ovary syndrome (PCOS), two conditions that significantly affect reproductive capacity in women (6). These metabolic disturbances create an unfavorable environment in the ovaries, leading to excessive androgen production, disruptions in lipid metabolism, and ultimately contributing to infertility (6, 7). Furthermore, PCOS is associated with a range of metabolic and reproductive manifestations, including infertility, obesity, IR, dyslipidemia, and glucose intolerance, all of which can result in long-term physiological and reproductive health complications in affected women (8, 9).

Dietary habits, both quantitative and qualitative, also seem to be linked to female infertility (5). For example, high-fat diets have been associated with poor oocyte development, apparently due to the induction of oxidative stress, particularly through the overexpression of genes like Nrf2 and SOD2, which regulate the oxidative stress response in the follicular environment (10). Specifically, several studies have shown that trans fatty acids (TFA) increase inflammatory biomarkers and IR (10, 11) and are associated with anovulatory infertility (12). The quantity and quality of carbohydrates can also influence reproductive health, as high-glycemic index products may increase IR, dyslipidemia, and promote oxidative stress, all of which negatively affect ovarian function (1, 12). Additionally, diets high in refined carbohydrates can exacerbate inflammatory responses, further contributing to reproductive challenges and fertility issues (12). Furthermore, the source of proteins appears to be an influential factor in female fertility. It has been observed that while animal protein intake seems to be linked to a higher risk of infertility, the consumption of plant-based proteins has positive effects on reproductive success (13). In a cohort study that included 18,555 women, it was found that consuming 5% of total energy intake as plant protein instead of animal protein was associated with a significantly reduced risk of ovulatory infertility (*p* = 0.007) (13).

In this regard, several studies have analyzed the effect of dietary patterns on female fertility. A study conducted in Spain concluded that among women attempting to conceive naturally, a high adherence to the Mediterranean Diet (MD) was associated with a shorter time to conception (14). The MD is characterized by a high intake of fruits, vegetables, whole grains, legumes, and nuts, with moderate consumption of fish and poultry, and low intake of red meat and dairy products (14). Similarly, according to the Nurse Health Study Cohort, adherence to a “pro-fertility diet” reduced the attributable risk of ovulatory disorder-related infertility by 66% (15). This diet was characterized by reduced intake of TFA and high consumption of plant-based protein and low-glycemic index, fiber-rich carbohydrates (15). However, the evidence in this regard is still limited, and some authors indicate that more information is needed to provide guidelines for the nutritional management of women of childbearing age (3).

The hypothesis of this study is that maintaining an optimal nutritional status, characterized by an adequate body weight, higher muscle mass, and a healthy distribution of body fat, along with a healthy diet high in plant-based proteins, reduces the risk of infertility in women of childbearing age. The objective of this study was to analyze how these specific nutritional factors and dietary habits influence the development of infertility.

## 2 Materials and methods

### 2.1 Study design and data collection

A case–control study was conducted in a sample of 90 women aged 18–40 years residing in the province of Alicante, Spain. In the case group (*n* = 60), women who attended the Gynecology-Sterility consultation of various hospitals after being referred by their primary care physician due to sterility of more than 1 year of evolution, or after 6 months if the woman was over 35 years old, were included. The exclusion criteria for the case group were: (1) women with a live and healthy child, (2) women with voluntary sterility, (3) women with documented medical conditions that contraindicate gestation or sterility treatment, (4) women with a documented situation related to any other circumstance that could severely interfere with the development of offspring, subject to the consideration of an ethics committee or similar body, (5) infertility due to male factors.

The control group (*n* = 30) was comprised of women who had just received a pregnancy diagnosis in various health centers and hospitals in the same province. To be included in the study, women could not exceed the 7th week of gestation. The exclusion criteria for the control group were: (1) women undergoing assisted reproduction techniques, (2) women who had experienced recurrent spontaneous abortions, with a history of infertility or previous conception problems.

Data collection was conducted from March 2023 to March 2024. The sample size had a power of 80%, with a type I error significance of 0.05. All participants completed a sociodemographic questionnaire (age, nationality, education level, employment status, annual income, etc.) and another questionnaire on adherence to the MD. Clinical and anthropometric variables were collected by trained personnel from the Gynecology service.

### 2.2 Anthropometric variables

The measurement of anthropometric variables was carried out by trained personnel following standardized methods. Body weight was determined using an OMRON digital scale, model HBF-212-EW, clinically validated with impedance, and values were recorded in kilograms (kg). Height was measured in centimeters (cm) using a vertical stadiometer, with an accuracy of 0.2 cm. Body Mass Index (BMI) was calculated using weight and height data (BMI = weight/height^2) and categorized according to current WHO guidelines (16).

To assess body composition, the same scale was used to measure body fat percentage (BFP), visceral fat percentage (VFP), and muscle mass percentage (MMP). A BFP of 33% or higher was considered indicative of excess fat mass, a VFP of seven or higher indicated cardiovascular risk (CVR), and MM*p* values below 30% were considered insufficient muscle mass (17). Additionally, waist and hip circumferences were determined using a SECA 201 measuring tape, and measurements were taken three times, with the average of the three measurements being recorded. Waist circumference was measured below the rib cage and above the navel, representing the narrowest part of the waist. Hip circumference was measured horizontally at the widest part of the buttocks. Using these values, the Waist-to-Hip Ratio (WHR = waist/hip) was calculated, with a value of 0.85 or higher indicating CVR (17, 18).

### 2.3 Dietary habits and Mediterranean Diet adherence assessment

To evaluate the level of adherence to the MD, the Mediterranean Diet Adherence Screener (MEDAS) questionnaire was used. This 14-question questionnaire has been validated in the Spanish population and was designed by the PREDIMED research group. Each question in the questionnaire was scored +1 if the response was positive for the MD, and a value of 0 if otherwise (19).

The total score was obtained by summing the values assigned to each question, determining the degree of adherence to the MD. Two categories were established: a total score of seven or higher indicated an optimal degree of adherence, while a score below seven indicated low adherence. It is important to note that the 14 items of the questionnaire were also individually analyzed to gain a more detailed understanding of dietary habits.

### 2.4 Data analysis

After collecting all the data, a database was created to include all the study variables. Statistical analysis was carried out using SPSS Version 25 for Windows (SPSS Inc., Chicago, United States).

Quantitative variables (age, weight, BMI, waist and hip circumference, WHR, BFP, VFP, and MMP) were described based on the distribution of the data. When the variables followed a normal distribution, they were represented by the mean and standard deviation (SD), and independent t-tests were used for group comparisons. When the variables did not follow a normal distribution, they were represented by the median and interquartile range, and the Mann–Whitney U test was applied.

For qualitative variables (e.g., education level, employment status), data were summarized as frequencies and percentages. Comparisons between groups for these variables were performed using Chi-square (*χ*^2^) tests.

Additionally, the Odds Ratio (OR) was calculated to assess the relationship between food consumption and the risk of infertility, with a specific focus on adherence to the MD. All statistical tests were two-tailed, and a *p*-value of <0.05 was considered statistically significant for all analyses.

### 2.5 Ethics statement

The research protocol received approval from the Ethics Committee of the Alicante Institute for Health and Biomedical Research (ISABIAL). The committee approval number was PI2021/113. All patients received detailed information about the objectives and characteristics of the study, as well as their voluntary participation and the right to withdraw at any time without consequences. Informed consent was then obtained from all participants before their inclusion in the study.

## 3 Results

### 3.1 Sociodemographic characteristics

Table 1 presents the baseline sociodemographic and health characteristics of the sample. The average age of both groups was similar: 32.38 (±4.25) years in infertile women and 32.80 (±3.98) years in fertile women. Most participants in both groups were European, with 86.7% (*n* = 52) of infertile women and 96.7% (*n* = 29) of fertile women being European. Education level, employment status, and socioeconomic status were also comparable between groups. Additionally, the majority of participants in both groups reported no known health conditions, with 93.33% (*n* = 56) of infertile women and 96.7% (*n* = 29) of fertile women being free of chronic conditions.

**Table 1:** Baseline sociodemographic and health characteristics of the sample of fertile and infertile women in the province of Alicante (*n* = 90).

Sociodemographic data	Infertile women (*n* = 60)		Fertile women (*n* = 30)		*P-*value
	*n*	%	*n*	%	
Nationality					
European	52	86.70	29	96.70	0.262
American	8	13.30	1	3.30	
Employment status					
Employee	51	85.00	26	86.70	0.834
Unemployed	9	15.00	4	13.30	
Levels of education					
None or primary	2	3.30	2	6.70	0.692
Secondary	15	25.00	7	23.30	
Higher education	43	71.70	21	70.00	
Socioeconomic levels					
Low	18	30.00	10	33.33	0.903
Middle	35	58.30	16	53.33	
High	7	11.70	4	13.33	
Health status					
No known conditions	56	93.33	29	96.70	0.661
Chronic conditions	4	6.66	1	3.30	

### 3.2 Anthropometric characteristics

Significant differences in anthropometric characteristics were observed between fertile and infertile women, particularly in muscle mass percentage (*p* = 0.005) and hip circumference (*p* = 0.034). Fertile women had a higher muscle mass percentage (28.64 ± 4.22% vs. 25.62 ± 4.87%) and a smaller hip circumference (97.06 ± 10.64 cm vs. 103.39 ± 13.66 cm) compared to infertile women. Additionally, fertile women tended to have a lower body fat percentage (*p* = 0.057) and a smaller waist circumference (*p* = 0.058), although these differences were not statistically significant. Other anthropometric measures did not show significant differences (see Table 2).

**Table 2:** Anthropometric characteristics of the sample of fertile and infertile women in the province of Alicante (*n* = 90).

Anthropometric variables	Infertile women (*n* = 60)		Fertile women (*n* = 30)		*P-*value
	Mean	SD	Mean	SD	
BMI (kg/m^2^)	25.54	5.89	23.93	3.98	0.130
Body fat (%)	36.55	9.58	32.59	8.28	0.057
Visceral fat (%)	5.53	2.42	4.77	1.75	0.127
Muscle mass (%)	25.62	4.87	28.64	4.22	0.005*
Waist circumference (cm)	82.05	15.43	74.67	17.67	0.058
Hip circumference (cm)	103.39	13.66	97.06	10.64	0.034*
WHR	0.79	0.97	0.78	0.94	0.844

BMI, body mass index; WHR, waist-to-hip ratio; *, level of significance assumed at *p* < 0.05.

### 3.3 Association between MD adherence and infertility

No statistically significant difference was found in overall adherence to the MD between fertile and infertile women (*p* = 0.742), with similar MEDAS scores in both groups: 7.87 (±1.61) for fertile women and 7.58 (±2.20) for infertile women. However, when analyzing individual components of the MEDAS test, a significant association was found between preferential intake of red meat and infertility (*p* = 0.011). Infertile women showed a higher intake of red meat, with 25.0% (*n* = 15) consuming it preferentially, compared to only 3.3% (*n* = 1) of fertile women.

Additionally, consuming one or more servings of red meat per day was marginally associated with infertility (*p* = 0.051), with 36.7% (*n* = 22) of infertile women reporting this intake compared to 16.7% (*n* = 5) of fertile women (see Table 3 for further details).

**Table 3:** Relationship between the development of infertility and adherence to the Mediterranean Diet in a sample of women from the province of Alicante (*n* = 90).

MEDAS Test Questions	Cases (*n* = 60)		Controls (*n* = 30)		*P-*value
	*n*	%	*n*	%	
1. Do you use olive oil as the main fat for cooking?					
Yes	56	93.3	29	96.7	0.661
No	4	6.7	1	3.3	
2. How much olive oil do you consume in total per day (including that used for frying, eating out, salads, etc.)?					
4 or more tablespoons	28	46.7	10	33.3	0.227
Less than 4 tablespoons	32	53.3	20	66.7	
3. How many servings of vegetables do you consume per day? (side dishes = 1/2 serving) 1 serving = 200 g.					
2 or more per day	34	56.7	15	50.0	0.549
Less than 2 per day	26	43.3	15	50.0	
4. How many pieces of fruit (including natural juice) do you consume per day?					
3 or more per day	12	20.0	7	23.3	0.715
Less than 3 per day	48	80.0	23	76.7	
5. How many servings of red meat, hamburgers, sausages or processed meats do you consume per day? (serving: 100–150 g)					
Less than 1 per day	38	63.3	25	83.3	0.051
1 or more per day	22	36.7	5	16.7	
6. How many servings of butter, margarine, or cream do you consume per day? (individual portion: 12 g)					
Less than 1 per day	55	91.7	27	90.0	1.000
1 or more per day	5	8.3	3	10.0	
7. How many carbonated and/or sugary drinks (soft drinks, colas, tonics, etc.) do you consume per day?					
Less than 1 per day	50	83.3	27	90.0	0.532
1 or more per day	10	16.7	3	10.0	
8. Do you drink wine? How much do you consume per week?					
7 or more glasses per week	7	11.7	0	0.0	0.090
No	53	88.3	30	100.0	
9. How many servings of legumes do you consume per week? (1 plate or serving of 150 g)					
3 or more per week	14	23.3	8	26.7	0.729
Less than 3 per week	46	76.7	22	73.3	
10. How many servings of fish/seafood do you consume per week? (1 plate, piece or serving: 100–150 g of fish or 4–5 pieces or 200 g of seafood)					
3 or more per week	13	21.7	5	16.7	0.576
Less than 3 per week	47	78.3	25	83.3	
11. How often do you consume commercial (non-homemade) pastries such as cookies, flans, sweets, or cakes per week?					
Less than 2 per week	34	56.7	19	63.3	0.545
2 or more per week	26	43.3	11	36.7	
12. How often do you consume nuts per week? (serving: 30 g)					
3 or more per week	31	51.7	15	50.0	0.881
Less than 3 per week	29	48.3	15	50.0	
13. Do you preferably consume chicken, turkey or rabbit instead of beef, pork, hamburgers, or sausages? (chicken meat: 1 piece or serving of 100–150 g)					
Yes	45	75.0	29	96.7	0.011*
No	15	25.0	1	3.3	
14. How many times a week do you consume vegetables, pasta, rice or other dishes dressed with tomato sauce, garlic, onion, or leek slow-cooked with olive oil (sofrito)?					
2 or more per week	38	63.3	20	66.7	0.755
Less than 2 per week	2	36.7	10	33.3	

MEDAS, Mediterranean Diet Adherence Screener; *, level of significance assumed at *P* < 0.05.

The multivariable analysis identified two significant predictors of infertility: preferential red meat consumption and muscle mass percentage. Preferential consumption of red meat was positively associated with infertility (*B* = 0.297, 95% CI: 0.053–0.541, *p* = 0.018), while muscle mass was inversely associated with infertility (*B* = 0.026, 95% CI: 0.007–0.046, *p* = 0.008). The overall model was statistically significant (*p* < 0.001), with an *R*-squared of 0.144, indicating that these variables together accounted for 14.4% of the variance in infertility. The final model was simplified to include only these two significant predictors.

## 4 Discussion

The objective of this study was to analyze the relationship between nutritional status, dietary habits, and infertility in women from the province of Alicante. The main findings reflect that infertile women have lower muscle mass and a larger hip circumference compared to fertile women. Additionally, it was found that higher red meat consumption is significantly associated with an increased risk of infertility.

To date, research analyzing the association of nutritional status with female infertility has mainly focused on BMI, overlooking other anthropometric parameters. In this regard, a case–control study conducted by Rich-Edwards et al. (20) examined the relationship between BMI and infertility due to ovulatory disorders, finding that a high BMI was associated with an increased risk of female infertility. However, this study did not evaluate body composition in terms of muscle mass or fat distribution, aspects that can offer a more detailed perspective on the relationship between nutritional status and fertility. Similarly, a study by Van der Steeg et al. (21) explored the influence of BMI on female fertility and found that both underweight and overweight were associated with a lower probability of pregnancy in women with regular ovulatory cycles, but did not analyze other anthropometric parameters.

Regarding the effect of muscle mass on female fertility, a multicenter case–control study found that both high body fat (OR = 3.16, 95% CI: 1.36–7.37) and low lean mass (OR = 2.65, 95% CI: 1.10–6.37) were associated with female infertility (22). However, few studies have specifically investigated the relationship between muscle mass and fertility despite the relevance of this anthropometric parameter. Muscle mass is not only an indicator of strength and physical capacity but also of metabolic health. Lower muscle mass can be associated with decreased insulin sensitivity and alterations in glucose metabolism, conditions linked to reproductive problems (23). In addition, skeletal muscle influences hormonal regulation through the production of myokines, which can impact ovarian function (24).

In line with the observed muscle mass differences, the larger waist and hip circumference in infertile women may indicate fat distribution patterns associated with insulin resistance and hormonal imbalances. Abdominal fat, for instance, is known to contribute to an adverse metabolic profile, including elevated insulin and androgen levels, which can disrupt ovulation and fertility (25). Studies such as those by Wang et al. (26) and Ke et al. (27) reinforce these findings, with both identifying waist circumference as a key predictor of reduced fertility, independent of BMI. The pattern of fat distribution, particularly in the abdominal region, may also be linked to chronic inflammation, further complicating reproductive outcomes (27).

On the other hand, the association found between higher red meat consumption and infertility is consistent with previous studies linking high red meat intake with adverse effects on metabolic and reproductive health. In this regard, a study including 2,659 embryos recovered from 269 patients undergoing intracytoplasmic sperm injection cycles observed that red meat consumption negatively affected embryonic development (*p* = 0.049) and pregnancy probability (*p* = 0.042) (28). Similarly, a cohort study by Chavarro et al. (13) concluded that replacing animal protein with plant protein sources could reduce the risk of ovulatory infertility (*p* = 0.007). Red meat consumption, particularly in large quantities, increases the risk of conditions that contribute to female infertility. A meta-analysis pooling data from four studies revealed a significant association between red meat intake and the risk of endometriosis, with a pooled effect size of relative risk 1.17 (95% CI: 1.08–1.26; *p* < 0.001) (29). Moreover, red meat consumption has been linked to elevated levels of inflammatory biomarkers, which could contribute to fertility issues. Additionally, the high saturated fat content in red meat can contribute to IR and hormonal imbalances, exacerbating fertility issues (30). Another study found that diets rich in red and processed meat were associated with an unfavourable metabolic profile, including elevated levels of insulin and inflammatory markers (31, 32). These metabolic alterations can interfere with ovulation and reproductive function (30).

These findings underline the importance of considering not only body composition and dietary habits, but also physical activity in the evaluation and treatment of infertility. Interventions that promote muscle mass gain, localized fat reduction, and regular physical exercise can enhance metabolic health and reproductive outcomes. In addition, dietary modifications aimed at reducing red meat consumption and increasing the intake of plant-based foods are beneficial strategies for improving fertility. Future longitudinal studies are needed to evaluate how changes in nutritional status, dietary habits, and physical activity over time impact fertility. Moreover, further research should explore the combined effects of dietary and exercise interventions on reproductive health across diverse populations to validate these findings.

This study has some limitations that must be considered. Firstly, the sample size is not very large, which could limit the external validity of the results. However, the detailed analysis and rigorous selection of criteria for participant inclusion help mitigate this limitation and reinforce the reliability of the results. Secondly, the cross-sectional nature of the study prevents establishing definitive causal relationships between dietary factors and infertility. Therefore, it is recommended that future research focus on longitudinal studies that can evaluate changes in nutritional status and dietary habits over time in relation to fertility. Additionally, it would be beneficial to explore specific dietary interventions and their impact on reproductive health, as well as study these associations in diverse populations to observe if the results are consistent across different contexts.

Despite these limitations, this study presents several significant strengths. The case–control design used allows for clear associations between dietary factors and infertility. Furthermore, the use of standardized methods for the collection of anthropometric and dietary data adds robustness to the findings. Similarly, the inclusion of a wide range of dietary and anthropometric variables allows for a detailed analysis of multiple factors that could influence infertility, providing a more comprehensive view of the problem. These strengths increase the internal validity of the study and provide a solid basis for future research in this area.

## 5 Conclusion

This study has identified significant associations between nutritional status, dietary habits, and female infertility. Specifically, it found that reduced muscle mass and larger hip circumference are associated with an increased risk of infertility. Additionally, frequent consumption of red meat is linked to a higher likelihood of female infertility.

These results highlight the need for a comprehensive approach to preventing and treating female infertility, considering both body composition and dietary habits. Although the study’s cross-sectional design and limited sample size constrain the ability to generalize the findings, they provide a valuable basis for future research. Subsequent studies should utilize longitudinal methodologies and broader demographic samples to further explore these relationships.

## Data Availability

The raw data supporting the conclusions of this article will be made available by the authors, without undue reservation.
